# 

**Published:** 1918-08

**Authors:** 


### ANNOUNCEMENTS

---

### Iowa State Board

The next meeting of the Iowa State Board for the examination of applicants will be held in Iowa City, Iowa, commencing August 26th at 9:00 a. m. For further information address the secretary. Dr. J. A. West, 417 Utica Bldg., Des Moines, Iowa.

---

### United States Employment Service

*Editor* Dental Register: We beg to direct your attention to the plans of the United States Employment Service, and to the great effect which this program will have upon the industrial life of the Nation.

On August 1, the supplying of war industries with common labor will be centralized in the United States Employment Service of the Department of Labor, and all independent recruiting of common labor by manufacturers having a payroll of more than 100 men will be diverted to the United States Employment Service. This is in accordance with the decision of the War Labor Policies Board and approved by the President on June 17. (The War Labor Policies Board is composed of representatives of the War, Navy and Agricultural Departments, the Shipping Board and the Emergency Fleet Corporation, the War Industries Board, and the Food, Fuel and Railroad Administrations. Its chairman is Felix Frankfurter, Assistant to the Secretary of Labor.)

The above action was found necessary to overcome a perilous shortage of unskilled labor in war industries. This shortage was aggravated by an almost universal practice of labor stealing and poaching.

While the restrictions against the private employment of labor apply only to common labor at the present time, these restrictions will, as soon as possible, be extended to include skilled labor. In the meantime, recruiting of skilled labor for war production will be subject to federal regulations now being prepared.

This drastic change in the Nation's labor program has been found necessary in order to protect the employer and the employed, to conserve the labor supply of the communities and to cut down unnecessary and expensive labor turn-over (which, in some cases, is as high as 100 per cent. a week), and to increase the production of essentials.

While non-essential industries will be drawn upon to supply the necessary labor for war work, the withdrawal will be conducted on an equitable basis in order to protect the individual employer as much as possible.

Under the operating methods adopted, the country has been divided into thirteen federal districts, each district in charge of a superintendent of the United States Employment Service. The states within each district are in turn in charge of a State Director, who has full control of the service within his state.

In each community there is being formed a local community labor board, consisting of a representative of the United States Employment Service, a representative of employers and a representative of the employed. This board will have jurisdiction over recruiting and distributing labor in its locality.

A survey of the labor requirements is being made, and in order that each community may be fully protected, rulings have been issued that no labor shall be transported out of any community by the United States Employment

Service without the approval of the State Director; nor shall any labor be removed by the Service from one state to another without the approval of the United States Employment Service at Washington. Every effort will be made to discourage any movements from community to community or state to state by any other service.

This labor program has the approval of all producing departments of the Government, through the War Labor Policies Board.

It must be understood that farm labor will be protected, for the industrial program distinctly includes special efforts to keep the farmer supplied with labor.

The requirement that unskilled labor must be recruited through the sole agency of the United States Employment Service does not at present apply in the following five cases:

1. Labor which is not directly or indirectly solicited.
2. Labor for the railroads.
3. Farm labor—to be recruited in accordance with existing arrangement with Department of Agriculture.
4. Labor for non-war work.
5. Labor for establishments whose maximum force does not exceed one hundred.

When the survey of labor requirements has been made and the aggregate demand for unskilled labor in war work is found, each state will be assigned a quota, representing the common labor to be drawn from among men engaged in non-essential industries in that state.

These state quotas will in turn be distributed among localities. Within each locality, employers in non-war work, including those who are only partially in war work, will be asked to distribute the local quotas from time to time amongst themselves. Quotas by localities and individuals are to be accepted as readily as they are for Liberty Loan and Red Cross campaigns. This plan of lauor quotas is a protection for all communities.

The object is to keep any community from being drained of labor, and to use local supply, as far as possible, for local demand. The situation, however, is such that in certain cases some men may have to be transported over long distances.

You will note from the above outline that this is probably the most drastic action that the Government has taken since putting the National Army draft into effect. The absolute necessity for this program can be seen when it is realized that in Pittsburgh, for instance, there are advertisements calling for men to go to Detroit; while in Detroit street cars there are posters asking men to go to Pittsburgh. This same condition is apparent all over the United States and in the consequent shifting of labor a great part of our war effort is dissipated.

Because this is one of the greatest problems facing the Nation today, we are asking that you give this matter your careful consideration. You will probably desire to carry some comment on this basic change in the Nation's labor methods, and we would suggest that if you desire to assign one of your men to look into this situation, the facilities of the Department of Labor and the United States Employment Service are at your disposal. Respectfully, J. B. Densmore, Director General, Department of Labor.

---

### New Naval Legislation

The following is taken from the conference report of the Senate and House Committee as its report on the Naval appropriation for Dentistry. Report No. 696. 2d Session, 65th Congress.

That the Act approved August twenty-ninth, nineteen hundred and sixteen, entitled "An Act making appropriations for the naval service for the fiscal year ending June thirtieth, nineteen hundred and seventeen, and for other purposes" (Statutes at Large, volume thirty-nine, chapter four hundred and seventeen, pages five hundred and seventy-three and five hundred and seventy-four), be, and the same is hereby, amended by striking out all of said Act following the caption "Naval Dental Corps" on page five hundred and seventy-three, but preceding the caption "Dental Reserve Corps," on page five hundred and seventy-four, and by substituting therefor the following:

"That the President of the United States is hereby authorized to appoint and commission, by and with the advice and consent of the Senate, dental officers in the Navy at the rate of one for each thousand of the total authorized number of officers and enlisted men of the Navy and Marine Corps, in the grades of assistant dental surgeon, passed assistant dental surgeon and dental surgeon, who shall constitute the Naval Dental Corps, and shall be a part of the Medical Department of the Navy. Original appointments to the Naval Dental Corps shall be made in the grade of assistant dental surgeon with the rank of lieutenant (junior grade), and all dental officers now in the Dental Corps appointed under the provisions of the Act of Congress approved August twenty-second, nineteen hundred and twelve (Statutes at Large, volume thirty-seven, page three hundred and forty-five), or under the provisions of the Act of Congress approved August twenty-ninth, nineteen hundred and sixteen (Statutes at Large, volume thirty-nine, page five hundred and seventy-three), or who may hereafter be appointed, shall take rank and precedence with officers of the Naval Medical Corps of the same rank according to the dates of their respective commissions or original appointments, and all such dental officers shall be eligible for advancement in grade and rank in the same manner and under the same conditions as officers of the Naval Medical Corps with or next after whom they take precedence, and shall receive the same pay and allowances as officers of corresponding rank and length of service in the Naval Medical Corps up to and including the rank of lieutenant commander: Provided, That dental surgeons shall be eligible for advancement in pay and allowances, but not in rank, to and including the pay and allowances of commander and captain, subject to such examinations as the Secretary of the Navy may prescribe, except that the number of dental surgeons with the pay and allowances of captain shall not exceed four and one-half per centum and the number of dental surgeons with the pay and allowances of commander shall not exceed eight per centum of the total authorized number of dental officers: Provided further, That dental surgeons shall be eligible for advancement to the pay and allowances of commander and captain when their total active service as dental officers in the Navy is such that if rendered as officers of the Naval Medical Corps, it would place them in the list of medical officers with the pay and allowances of commander or captain, as the case may be. And provided further, That dental officers who shall have gained or lost numbers on the Navy list shall be considered to have gained or lost service accordingly; and the time served by dental officers on active duty as acting assistant dental surgeons and assistant dental surgeons under provisions of law existing prior to the passage of this Act shall be reckoned in computing the increased service pay and service for precedence and promotion of dental officers herein authorized or heretofore appointed.

''All appointees authorized by this Act shall be citizens of the United States between twenty-one and thirty-two years of age, and shall be graduates of standard medical or dental colleges and trained in the several branches of dentistry, and shall, before appointment, have successfully passed mental, moral, physical and professional examinations before medical and professional examining boards appointed by the Secretary of the Navy, and have been recommended for appointment by such boards: Provided, That hereafter no person shall be appointed as assistant dental surgeon in the Navy who is not a graduate of a standard medical or dental college.

''Officers of the Naval Dental Corps shall become eligible for retirement in the same manner and under the same conditions as now prescribed by law for officers of the Naval Medical Corps, except that section fourteen hundred and forty-five of the Revised Statutes of the United States shall not be applicable to dental officers, and they shall not be entitled to rank above lieutenant commander on the retired list, or to retired pay above that of captain.

''All dental officers now serving under probationary appointments shall become immediately eligible for permanent appointment under the provisions of this Act, subject to the examinations prescribed by the Secretary of the Navy for original appointment as dental officers, and may be appointed assistant dental surgeon with the rank of lieutenant (junior grade) to rank from the date of their probationary appointments: Provided, That the senior dental officer now at the United States Naval Academy shall not be displaced by the provisions of this Act, and he shall hereafter have the grade of dental surgeon and the rank, pay, and allowances of lieutenant commander, and he shall not be eligible for retirement before he has reached the age of seventy years, except for physical disability incurred in the line of duty: Provided further, That no dental officer in the Navy who on original appointment as dental officer was over forty years of age shall be eligible for retirement before he has reached the age of seventy years, except for physical disability incurrd in line of duty.

''All Acts or parts of Acts inconsistent with the provisions of this Act relating to the Dental Corps of the Navy are hereby repealed: Provided, That nothing herein contained shall be construed to legislate out of the service any officer now in the Medical Department of the Navy or to reduce the rank, pay, or allowances now authorized by law for any officer of the Navy.

''That all laws heretofore enacted by Congress relating to the Medical and Dental Corps be, and the same hereby are repealed: Provided, That members of the Medical Reserve Corps and Dental Reserve Corps may be enrolled in the Naval Reserve Force in the present grades and ranks."

---

### Preparedness League of American Dentists—Communication from the President

*Free Dental Service.* There are a few points about free dental service that should be emphasized at this time.

1. The League requests its members to give each drafted man *one hour* of free dental service. This should be given without question as to ability to pay.

When the hour has been completed, the dentist is at liberty to inquire as to the ability of the registrant to pay a fee to have the work completed, should additional service be required. Should he prove himself worthy of further free service, the dentist continues such service in the *name of the League.*

*Such service only, is reported to the League.*

2. All dental work for which a fee is received from the drafted man *is not Preparedness League work.*

*Such service must not be reported to the League.*

Free is a word that can not be qualified and admits of no variation. The charge of a single farthing nullifies the spirit of the League through which free dental service is given.

We have a million of our boys in France. How many of them are suffering from dental ills because you and I did not give them free dental service? Thousands are suffering today because you and I did not do our full duty by them. It is a serious matter and let us resolve to throw our best efforts into this great work which we alone can do.

Let us fight the enemy with our own weapons—those with which we are most familiar. As dentists, each one of us can do more to help win the war with the excavator, the engine and the forceps than we could by shouldering a gun and taking a place in the first line trenches.

Each one of us can do this in our own offices by giving free dental service to the drafted man. Give it freely and without restraint as one of the great privileges of an American citizen and let us all be thankful that we are able to do this through the heritage of the greatest birthright given by any nation.

President Wilson has shown us our duty when he said: "An army can win a battle, but it takes a Nation to win a war."

I commend to the attention of every member of the League the following letter, which concisely covers the situation and may be considered a rule to follow. It is most essential that we conform in every particular to the ruling of the War Department:

*War Department, Office of the Provost Marshal General, Washington,*

June 8, 1918.

Dr. J. W. Beach, President Preparedness League of American Dentists, Buffalo, N. Y.

*Dear Sir:* This is to acknowledge your favor of June 4th, and to say that, it together with enclosures, has been carefully noted. This office very much appreciates the incalculable value of the work your League is doing and the prompt and full co-operation of its officers with the Government.

In the course of the enormous work you are doing, misunderstandings have necessarily arisen between dentists and the registrants for whom they were voluntarily working, but such misunderstandings are by no means confined to the profession of dentistry. We realize that some who are able and ought to pay for dental services rendered will try to get service without payment. This, of course, is not contemplated by any fair-minded person, much less by the Government. Registrants who are able to pay for the necessary work ought to do so, and those demanding very expensive work, or more than is necessary to fit them for military service, ought at least to pay the expense.

In order, however, to avoid any misunderstandings, and to prevent any possibility of abuse or scandal, I think there should be no attempt whatever to differentiate between those who may and those who may not be able to pay, so far as concerns the actual work necessary to fit for military duty. I think the rule ought to be clearly and fully announced, that *any registrant who is sent to one of your members and presents himself for treatment comes as a result of a statement that the work is to be done without cost to him,* just as if he were already in the service and were being treated by Army Surgeons, and is to be given such treatment as may be necessary, without any thought or inquiry as to whether or not he is able to pay. If he voluntarily and without demand offers to pay what he can, or if he requests additional treatment beyond what is absolutely essential, that is a matter of agreement between him and the dentist.

I hope I have made myself perfectly clear, and am sure that you agree with the proposition I have advanced.

I hope that you will convey to the members of the League the assurance that the value of the magnificent, patriotic work it has undertaken and is performing is fully appreciated not only by this office and by the War Department, but also by all our people to whom it is day by day becoming better known. Very truly yours, (Signed) J. S. Easby-Smith, Lieut-Colonel, N. A., Chief, Law Division.

The Treasurer has from time to time received requests from League workers in various parts of the country, for from fifty to five hundred buttons to be sent for distribution at state or local meetings. Because of action taken at a meeting of the officers held at League Headquarters in January, President Beach presiding, to the effect that buttons should be sent direct from the Treasurer's office and shall serve as his receipt, the Treasurer has had no alternative but to abide by the ruling. It is hoped that his position has been understood by those who made requests and that his letter of explanation met with their approval. It is obvious that if allotments of buttons were sent to many meetings in various sections of the country, the

League would be compelled to have an investment in buttons amounting to several thousands of dollars. This the funds will not permit.

There is also another serious objection. The button is supplied gratis to members who have joined since the first of January, 1918. To those who joined before, it is sold for twenty-five cents, which is approximately cost. Without a complete directory of the 5,900 older members, it is impossible for those handling the buttons at a meeting to know who is entitled to purchase them. Therefore, some men might be wearing the buttons who were really not members of the League. The officers I am sure might be glad to consider any feasible suggestion by which it would be possible to have buttons at the various meetings. There was delay in sending the buttons in February, because of disappointment in manufacture, but since that time buttons have been sent out regularly each week. Under the present plan, when the member receives a button, it is assured that his name has been registered on the official list.

The following figures show the amount of work done by the Preparedness League to June 1, 1918.

| | |
|---|---|
| Men examined | 84,518 |
| Men worked for and reported by card | 63,625 |
| Fillings inserted | 180,593 |
| Teeth extracted | 67,247 |
| Plates made | 630 |
| Crowns made | 2,040 |
| Crowns and bridges | 165 |
| Bridges | 885 |
| Prophylaxis treatments | 33,671 |
| Diseased teeth treated | 3,165 |
| Miscellaneous operations | 48,535 |
| Total operations performed | 336,931 |

The above report is taken from tabulated statistics made by Major W. A. Heckard.

